# Molecular Basis of the Therapeutical Potential of Clove (*Syzygium aromaticum* L.) and Clues to Its Anti-COVID-19 Utility

**DOI:** 10.3390/molecules26071880

**Published:** 2021-03-26

**Authors:** Caterina Vicidomini, Valentina Roviello, Giovanni N. Roviello

**Affiliations:** 1 Istituto di Biostrutture e Bioimmagini IBB-CNR, Via Tommaso De Amicis 95, 80145 Naples, Italy; caterina.vicidomini@ibb.cnr.it; 2 Department of Chemical Materials and Industrial Production Engineering (DICMaPI), University of Naples Federico II, Piazzale V. Tecchio 80, 80125 Naples, Italy; valentina.roviello@unina.it

**Keywords:** coronavirus infections, pandemics, natural compounds, clove, *Syzygium aromaticum*, eugenol, eugeniin, SARS-CoV-2, COVID-19, phytochemicals, herbal medicine

## Abstract

The current COronaVIrus Disease 19 (COVID-19) pandemic caused by SARS-CoV-2 infection is enormously affecting the worldwide health and economy. In the wait for an effective global immunization, the development of a specific therapeutic protocol to treat COVID-19 patients is clearly necessary as a short-term solution of the problem. Drug repurposing and herbal medicine represent two of the most explored strategies for an anti-COVID-19 drug discovery. Clove (*Syzygium aromaticum* L.) is a well-known culinary spice that has been used for centuries in folk medicine in many disorders. Interestingly, traditional medicines have used clove since ancient times to treat respiratory ailments, whilst clove ingredients show antiviral and anti-inflammatory properties. Other interesting features are the clove antithrombotic, immunostimulatory, and antibacterial effects. Thus, in this review, we discuss the potential role of clove in the frame of anti-COVID-19 therapy, focusing on the antiviral, anti-inflammatory, and antithrombotic effects of clove and its molecular constituents described in the scientific literature.

## 1. Introduction

Coronaviridae is a family of enveloped RNA viruses known as Coronaviruses (CoVs) that provoke infections in animals and humans [1,2,3,4,5,6]. Presently, seven human coronaviruses (HCoVs), commonly considered of zoonotic origin, are described in the scientific literature [7] that cause infections mainly associated with respiratory symptoms [8,9,10]. More in detail, HCoV-229E, HCoV-NL63, HCoV-OC43, and HCoV-HKU1 are “common cold” coronaviruses causing seasonal, usually mild, respiratory diseases [11,12]. Although, in most cases, these HCoVs do not lead to severe clinical symptoms, HCoV-NL63 and HCoV-HKU1 infections can provoke bronchiolitis and croup [13,14], whilst CoV 229E and OC43 can provoke pneumonia [15,16]. Nonetheless, three highly pathogenic HCoVs have emerged in the last two decades, i.e., Middle East Respiratory Syndrome (MERS)-CoV, Severe Acute Respiratory Syndrome (SARS)-CoV-1, and SARS-CoV-2 [17,18,19], which can lead to life-threatening pathologic events associated with the recent MERS, SARS, and the current COronaVIrus Disease 19 (COVID-19), which is causing enormous problems globally in both sanitary and socioeconomic terms [20]. SARS-CoV-1 and MERS-CoV are more lethal than SARS-CoV-2, but this latter is more transmissible, explaining the current pandemic status of COVID-19 [21]. In the first step of coronavirus infection, a specific molecular recognition between the virus particle, through the virus spike (S) protein, and the host cell takes place, involving different HCoV-specific receptors [22,23,24,25,26] that were identified for several CoVs and are considered one of the primary targets for anti-CoV biomedical strategies together with the SARS-CoV-2 main protease (M^pro^) [27,28]. The receptors for the “common cold” HCoVs are human aminopeptidase N (APN), associated with the infection from HCoV-229E, and 9-O-acetylated sialic acid (9-O-Ac-Sia), used by HCoV-OC43 and HCoV-HKU1. On the other hand, the receptor for HCoV-NL63, i.e., angiotensin-converting enzyme 2 (ACE2), is also common to the more pathogenic SARS-CoV-1 and SARS-CoV-2, whilst dipeptidyl peptidase 4 (DPP4) was associated with MERS-CoV [29,30]. Once intracellular, all HCoVs replicate their RNA with the consequent expression of the viral proteins needed for the production of new viral particles inside the infected cell [31]. As anticipated, four out of the seven HCoVs are associated with usually mild upper respiratory infections, whilst MERS-CoV and SARS-CoV-1 and -2 can cause lethal events [32]. This latter, first emerging in China at the end of 2019 [32], can lead to severe pneumonia and, being easily transmissible, has spread worldwide rapidly, leading the World Health Organization (WHO) to declare COVID-19 a pandemic [33]. Currently, there are more than two million deaths (2,566,793, as found in Worldometers.info [34] accessed on 3 March 2021) worldwide due to COVID-19, with enormous consequences for the public health and the economy worldwide [35,36,37]. While the whole world is fighting against COVID19 and awaits an effective mass immunization, the scientific community is devoting immense efforts toward developing specific therapies for the treatment of SARS-CoV-2 infection. Moreover, since inflammatory cytokine storms together with immune system impairment are commonly observed in patients with severe COVID-19, several research studies have highlighted the advantages of dual therapies with antiviral and anti-inflammatory benefits [38,39]. Due to the urgent need for such a pharmacological treatment, drug repurposing [40,41,42] and herbal medicine are two of the most considered anti-COVID-19 approaches [43,44,45,46,47]. In fact, several plants such as mulberry, tea, and Dragon’s Blood tree are known as remedies to treat respiratory ailments and for their anti-inflammatory and antithrombotic properties, which are useful aspects in the fight against COVID-19 [48,49,50,51,52].

## 2. Clove (*Syzygium aromaticum* L.) in Herbal Medicine and Its Active Constituents

*Syzygium aromaticum* L., also known as Eugenia caryophyllata L. [53], is an evergreen tree with sanguine flowers belonging to the family Myrtaceae that grows in tropical climates and has been widely used in Ayurveda and Chinese traditional medicines for over 2000 years. Arabic traders brought it to the Western world in the fourth century A.D., and in medieval Europe, it became very popular as a medicinal spice [54].

Indigenous to the Moluccas, this tree is cultivated in several countries of Asia and Africa, including India, Indonesia, Madagascar, Malaysia, Sri Lanka, and Zanzibar [55]. The dried flower bud of this plant is indicated by the English name “clove”, derived from the Latin word “clavus” (nail), as the shape resembles that of a small-sized nail. Cloves are currently used in three different forms, as whole dried buds (commonly referred to as “cloves”), ground spice, and essential oil. Though all forms share similar biomedically-relevant properties, they differ in the degree of potency, with the oil showing the highest potency and, thus, often being diluted with almond oil. Whole cloves, containing a good amount of oil in their interiors, are still endowed with a medium potency, whilst ground cloves are the least potent form, as, in this form, the spice generally loses most of the essential oil [54].

Cloves have long been used in both traditional medicine and for culinary purposes and serve to produce an essential oil known since ancient times in food flavorings, traditional medicine, and perfume production [53]. Even though cloves are mostly used as a nutritional spice for food in the Western world, in the past, they have constituted a remedy for a variety of health concerns, with the clove anesthetic (due to eugenol), stimulating, antimicrobial, antifungal, antiviral, and antiseptic properties having been known for centuries [54].

On the other hand, the clove essential oil finds applications in dental care, including the treatment of gum infections [56], burns [57], and respiratory and digestive disorders [56,58]. The previous literature studies also evidenced other remarkable properties, such as antiangiogenic [53,59], anticancer [53,56,58], antioxidant [60], anti-inflammatory [61], and antimutagenic activities [62].

The American Food and Drug Administration (FDA) agency has confirmed the safety of clove buds, clove oil, and some clove ingredients as a food supplement [63], while the WHO has established the acceptable daily uptake of cloves in humans at 2.5 mg/kg body weight [64].

The spice contains a good amount of minerals like magnesium, manganese, potassium, iron, and selenium [54]. Among the others, potassium as an important electrolyte of the cell and body fluids has a key role in the heart rate and blood pressure control [65], while manganese is used by the body as a cofactor for the antioxidant enzyme superoxide dismutase [66,67]. Additionally, cloves are a good source of beta carotene vitamin B1, vitamin B6, vitamin C, vitamin K, riboflavin, and vitamin A, used by the body for maintaining healthy mucus membranes and skin [68]. Noteworthy, vitamin C sustains a resistance against infectious agents [69] and is used by cells to scavenge harmful oxygen-free radicals [70].

Several research studies have been carried out to identify the main clove phytochemicals [71,72,73,74,75,76,77]. Dried clove buds contain ~20% essential oil, which is rich in eugenol, accounting for 70–90%. The other main phytochemicals isolated from clove essential oil include eugenyl acetate, β-caryophyllene, and several sesquiterpenes [53,78], including α-cubebene, α-copaene, and γ- and δ-cadinene [79]. Crategolic acid, vanillin, gallotannic acid, methyl salicylate, eugeniin, rhamnetin, kaempferol, eugenitin, oleanolic acid, methyl amyl ketone, methyl salicylate, α- and β-humulene, benzaldehyde, chavicol, and β-ylangene are present in lesser amounts [74]. In particular, eugenol and minor constituents like methyl salicylate and methyl amyl ketone are responsible for the characteristic pleasant aroma of cloves. The extraction of phytochemicals, achievable with high efficiency by presoaking and the liquid ammonia treatment of plant materials [80], in the case of cloves was realized with different operating conditions, including using supercritical CO_2_ [81]_._

### 2.1. Clove as Herbal Remedy for Respiratory Ailments

Traditional medicine uses cloves as respiratory aids, and in particular, the spice is one of the ingredients of teas used in tropical Asia to facilitate coughing [54]. Moreover, an aromatherapy procedure consisting of breathing in the aroma released from hot clove tea is another common way to use cloves for respiratory disorders like coughs, colds, asthma, bronchitis, and sinusitis [54]. Moreover, it is customary in Asia to chew cloves for treating soreness of throat and inflammation of the pharynx [54]. Chewing cloves after their thermal treatment is reported to bring relief from severe coughing [54]. Clove oil acts as an expectorant for treating respiratory disorders, including colds, bronchitis, cough, asthma, and upper-respiratory conditions [74]. In mixtures with honey, it helps in the case of chronic coughs and is mentioned to be specifically useful in the case of shortness of breath [82].

### 2.2. Anti-Inflammatory, Immunostimulatory, and Antithrombotic Properties of Cloves

Clove essential oil, often used in aromatherapy to treat inflammatory diseases, including arthritis and rheumatism [54], was found to have anti-inflammatory effects in animal models at doses of 0.05 and 0.20 mL/kg [83]. Interestingly, at this dosage, the anti-inflammatory effect of clove oil matches that of anti-inflammatory drugs like etodolac and indomethacin administered at 0.025 and 0.1, and 0.05 and 0.2 mL/kg doses, respectively [83]. The ethanol extracts of clove buds were also tested for anti-inflammatory effects at three doses (50, 100, and 200 mg/kg) in mice and Wistar rats using acetic acid-induced abdominal contractions in the former and formalin-induced hind paw edema in the latter animal models. The extract with an LD_50_ (50% Lethal Dose) of 565.7 mg/kg produced significant effects at all three doses, supporting the use of the clove extract in inflammatory conditions [84].

From a molecular point of view, clove buds contain flavonoids like β-caryophyllene, kaempferol, and rhamnetin, which contribute to clove anti-inflammatory properties [85,86,87,88,89]. In experimental animal models, eugenol (at 200 and 400 mg/kg doses) was shown to reduce the volume of pleural exudates without changing the total count of blood leukocytes, which indicates the anti-inflammatory activity of this molecule [90]. Eugenol is believed to regulate the cellular inflammatory cascades, including the NF-κB (nuclear factor kappa-light-chain-enhancer of activated B cells) and ERK (extracellular-signal-regulated kinase)/MAPK (mitogen-activated protein kinase) pathways, and the release of proinflammatory interleukins [82]. In other studies, LPS (lipopolysaccharide)-induced lung inflammation was relieved by the treatment with both whole clove aqueous extract and eugenol through a reduction of TNF-α (tumor necrosis factor alpha) and inhibition of NF-κB signaling, also with improvement in the alveolar damage [91,92]. Remarkably, clove aqueous extract showed protective effects on an animal model of pyelonephritis [93], a kidney inflammation reported in COVID-19 patients [94].

Traditional medicine attributes to clove the property of boosting the human immune system, improving disease resistance [54]. In experimental studies on animal models, clove oil improved the total white blood cell count and enhanced the delayed-type hypersensitivity response. Noteworthy, a dose-dependent restoration of both humoral and cellular immune responses was observed in cyclophosphamide-immunosuppressed mice treated with clove essential oil. The immunostimulatory activity was associated with improvement in the cell- and humor-mediated immune response mechanisms determined by clove essential oil [95].

Clove is mentioned to improve the blood supply to both the brain and the heart and is used as a tonic for the cardiovascular system [82]. Moreover, clove oil was shown to inhibit the platelet aggregation induced by the platelet-activating factor, arachidonic acid, and collagen, with a higher activity observed in the first two systems than the latter [74]. In vivo experiments carried out on rabbits showed that clove oil at 50–100 mg/kg doses afforded total protection against the platelet-activating factor and good (70%) protection against arachidonic acid-induced shock due to pulmonary platelet thrombosis [74]. Clove oil also inhibited thromboxane-A2 and 12-hydroxyeicosatetraenoic acid production by human platelets treated with C-14 arachidonic acid [96]. Antithrombotic and antiplatelet aggregation effects were also studied on clove extracts by ex vivo methods measuring the fibrinolytic activity and the inhibitory effect on thrombin-induced platelet aggregation [97]. The extracts showed remarkable fibrinolytic activity and inhibitory effects on platelet aggregation, suggesting clove anti-atherosclerotic potential [97].

**Figure 1:**
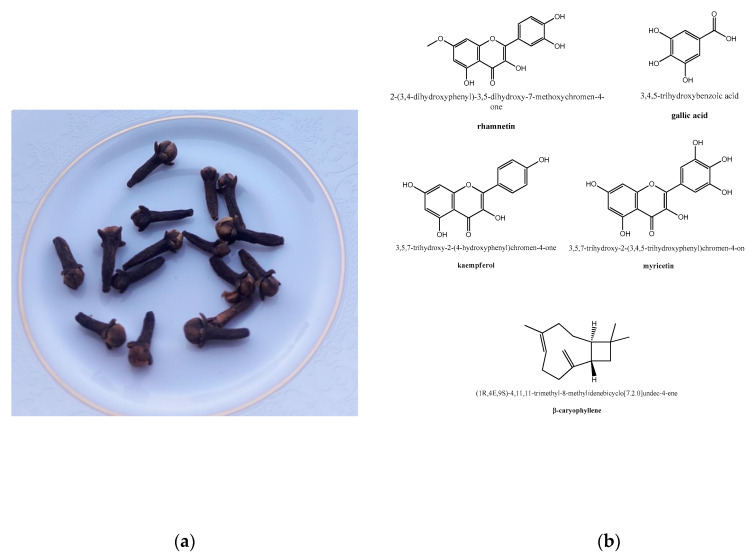
Clove, from culinary use to herbal medicine: (**a**) edible clove buds (photo taken by Giovanni N. Roviello). (**b**) Structure representation of some phytochemicals extracted from *Syzygium aromaticum* endowed with anti-inflammatory properties.

Owing to the molecular basis for the clove antithrombotic effects, the main clove oil constituent, eugenol, has shown activity as a platelet inhibitor, thus preventing blood clots [87]. More in detail, the same compound was shown in vitro to inhibit arachidonic acid-induced platelet aggregation, as well prostaglandin biosynthesis and the formation of thromboxane B2 [98]. Together with acetyl eugenol, it was more effective than acetylsalicylic acid in inhibiting the platelet aggregation induced by arachidonic acid, adrenaline, and collagen, showing, in the first case, an anti-aggregation activity comparable to indomethacin [99]. Aside from the above-mentioned antithrombotic properties of eugenol, these were also revealed for rhamnetin, gallic acid, kaempferol, myricetin, and β-caryophyllene (Figure 1), as well for two polysaccharides isolated from the clove buds by chromatographic methods [100].

Both polysaccharides presented a backbone of type I rhamnogalacturonan and the side chain made of arabinan. However, one mainly composed of the sugars Ara, Gal, Glc, and Rha was endowed with a relatively high molecular weight (MW ~103,000), and the other mainly composed of Rha, Gal, GalA, and Ara showed a lower molecular weight (MW ~34,000). The high molecular weight polysaccharide showed antithrombotic activity with a plasma clotting time of 145 s in the activated partial thromboplastin time (APTT) assays, while the other displayed a lower activity with a plasma clotting time of 90 s in the APTT assay [100].

## 3. Clove Antiviral Properties

The whole clove antiviral activity was tested by Tragoolpua and Jatisatienr [101], who assayed an ethanol extract obtained from the plant flower buds for its anti-herpes simplex virus (HSV) properties. By a plaque reduction assay, the authors demonstrated that HSV was inhibited by the clove extract. Interestingly, the clove extract showed a direct inactivating action on the particles of the standard HSV strains. Moreover, the total HSV virus yield at 30 h declined after the treatment with the extract [101]. Another study performed on the methanol extracts of cloves showed a high in vitro activity of the extract in inhibiting the HCV protease, with a ≥90% protease inhibition at a dose of 100 µg/mL [102].

### Antiviral Properties of Clove Phytochemicals

**Figure 2:**
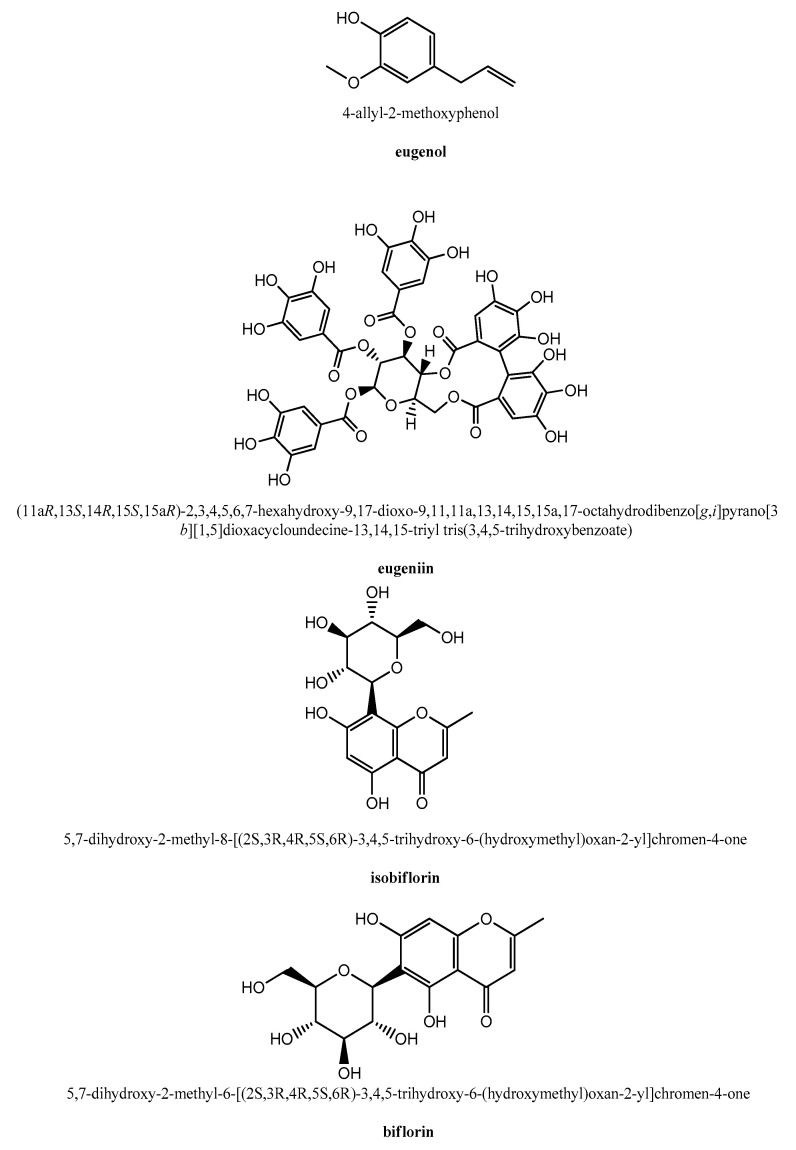
Structure representations of some clove phytochemicals with antiviral activity.

Eugenol (4-allyl-2-methoxyphenol; Figure 2), being the major constituent of cloves, was investigated for its antiviral activity by several research groups. The above-mentioned Tragoolpua and Jatisatienr [101] used pure eugenol as the reference compound in their anti-HSV studies and found that it exerted a higher antiviral activity than the ethanol extracts of whole clove buds. Similar findings were obtained by Benencia and Courreges [103], who reported the eugenol inhibition of HSV-1 and HSV-2 replication with inhibitory concentration 50% (IC_50_) values of 25.6 µg/mL and 16.2 µg/mL, respectively. In the same study, eugenol was virucidal, whilst no compound-associated cytotoxicity was revealed at the concentrations tested [103]. Eugenol also showed antiviral activity against the influenza A virus (IAV), being able to inhibit IAV replication [104]. Finally, it was also found active as an inhibitor of the Ebola Virus in vitro [105].

Other clove phytochemicals were investigated for their antiviral properties, and among them, eugeniin (Figure 2), isolated from the herbal extracts of cloves and, also, from *Geum japonicum*, showed anti-HSV activity at a 5-μg/mL concentration [106]. The HSV inhibitory activity of eugeniin was due to the inhibition of the viral DNA synthesis, as it acted as a selective inhibitor of the DNA polymerases of HSV-1 and HSV-2 [106].

Eugeniin was also found to act as a potent inhibitor of the protease of Dengue virus (DENV), which causes infections in tropical and subtropical regions of the world for which there are still no specific antiviral treatments available [107]. The IC_50_ values of eugeniin against the proteases of DENV serotype-2 and -3 were 94.7 nM and 7.5 μM, respectively. Thus, in consideration of the importance of DENV protease for the viral replication cycle, eugeniin was proposed as a promising drug in the context of anti-DENV therapeutics development [107]. The other investigated DENV protease inhibitors were isobiflorin and biflorin (Figure 2), even though their inhibitory activity was weaker than eugeniin [107]. The atomic-level details of the binding of these three clove phytochemicals to the viral protease were obtained by computational docking and saturation transfer difference (STD) NMR spectroscopy, which showed that the molecular recognition at the active site of the DENV protease involved networks of hydrophobic contacts and hydrogen bonds [107].

## 4. Clove in the Fight against COVID-19

The traditional therapeutic use of clove in respiratory disorders and its activity against different types of viruses, alongside its anti-inflammatory, immunostimulatory, and antithrombotic properties, are all attractive features highlighting its potential in the fight against the COVID-19 disease.

Clove is one of the medicinal plants currently employed to prevent and control the SARS-CoV-2-associated disease, together with *Eucalyptus globulus*, *Cymbopogon citratus*, *Zingiber officinale*, and other plants endowed with the advantage of being inexpensive and abundantly available around the globe [108]. More in detail, a protocol for the prevention and treatment of COVID-19 using cloves, as medicinal plant, was described by Kanyinda, J.N. M., who reported a proven effect for the treatment provided that it was carried out in the early stages of the disease [108]. The protocol included the preparation of a decoction in which cloves are boiled in water with other plant materials for 15 min. The released volatile active principles are then inhaled by patients for five minutes. The same protocol also included a drinkable decoction obtained with cloves and other plant materials [108]. Noteworthy, surveys have been conducted in India and Morocco, countries with low pandemic impacts [109,110], to identify the various home remedies used by the local populations during COVID-19, which have included many spices and herbs. Interestingly, more than 93% of the interviewed Indian people believed that spices are helpful in curing COVID-19 or other viral infections and can help in boosting the immunity. Cloves are mentioned as one of the most frequently used spices and herbs during the current COVID-19 pandemic in the areas under investigation, together with other plants like cinnamon, ginger, black pepper, garlic, neem, and basil [111]. Cloves are also being used in Morocco by herbalists from Salé Prefecture for the prevention and treatment of COVID-19 [112]. From a molecular point of view, some computational studies recommended phytocompounds extracted from cloves as potent anti-COVID-19 drugs [113,114], and one of them, kaempferol, was shown in silico to bind the substrate binding pocket of the main protease of SARS-CoV-2 with high affinity interacting with the active site residues such as Cys145 and His41 through hydrophobic interactions and hydrogen bonding, suggesting that natural compounds such as clove flavonoids could act as novel inhibitors of SARS-CoV-2 [115]. Molecular docking studies have also shown high affinities of clove compounds bicornin (−9.2 kcal/mol) and biflorin (−8.5 kcal/mol) for M^pro^, suggesting their potential inhibitory activity [115].

## 5. Conclusions

The therapeutic use of cloves in traditional medicine to treat respiratory ailments and its experimentally proven activity against different types of viruses, as well its anti-inflammatory, immunostimulatory, and antithrombotic properties, all concur to compose a picture of the potential importance of cloves and their phytochemical constituents in the fight against the COVID-19 disease. Aside from the above-mentioned features, clove essential oil has shown remarkable antibacterial effects against the infections of immunosuppressed hospitalized patients [78], suggesting its utility to also prevent secondary bacterial infections in COVID-19 patients [82]. In conclusion, cloves, a precious spice largely used in countries where the impact of the novel coronavirus is lower than the Western world, are endowed with medicinal properties considered relevant in the prevention and therapy of COVID-19. Future clinical data on the activity of cloves and their constituents on COVID-19 patients and more molecular insights on the specific clove phytochemical interactions with SARS-CoV-2 protein targets are clearly desirable in order to realize the effective therapeutic protocols and design new drugs based on clove phytochemicals with optimized characteristics.

## References

[1] V’kovski P., Kratzel A., Steiner S., Stalder H., Thiel V. (2020). Coronavirus biology and replication: Implications for SARS-CoV-2. Nat. Rev. Microbiol..

[2] Chu D.K.W., Leung C.Y.H., Gilbert M., Joyner P.H., Ng E.M., Tse T.M., Guan Y., Peiris J.S.M., Poon L.L.M. (2011). Avian Coronavirus in wild aquatic birds. J. Virol..

[3] Poon L.L.M., Chu D.K.W., Chan K.H., Wong O.K., Ellis T.M., Leung Y.H.C., Lau S.K.P., Woo P.C.Y., Suen K.Y., Yuen K.Y. (2005). Identification of a novel coronavirus in bats. J. Virol..

[4] Wang L.-F., Anderson D.E. (2019). Viruses in bats and potential spillover to animals and humans. Curr. Opin. Virol..

[5] Memish Z.A., Mishra N., Olival K.J., Fagbo S.F., Kapoor V., Epstein J.H., AlHakeem R., Durosinloun A., Al Asmari M., Islam A. (2013). Middle East respiratory syndrome coronavirus in bats, Saudi Arabia. Emerg. Infect. Dis..

[6] Hofmann H., Pyrc K., van der Hoek L., Geier M., Berkhout B., Pohlmann S. (2005). Human coronavirus NL63 employs the severe acute respiratory syndrome coronavirus receptor for cellular entry. Proc. Natl. Acad. Sci. USA.

[7] Ye Z.-W., Yuan S., Yuen K.-S., Fung S.-Y., Chan C.-P., Jin D.-Y. (2020). Zoonotic origins of human coronaviruses. Int. J. Biol. Sci..

[8] Gossner C., Danielson N., Gervelmeyer A., Berthe F., Faye B., Kaasik Aaslav K., Adlhoch C., Zeller H., Penttinen P., Coulombier D. (2014). Human–dromedary camel interactions and the risk of acquiring zoonotic middle east respiratory syndrome Coronavirus infection. Zoonoses Public Health.

[9] Sheahan T., Rockx B., Donaldson E., Sims A., Pickles R., Corti D., Baric R. (2008). Mechanisms of zoonotic severe acute respiratory syndrome coronavirus host range expansion in human airway epithelium. J. Virol..

[10] Sheahan T., Rockx B., Donaldson E., Corti D., Baric R. (2008). Pathways of cross-species transmission of synthetically reconstructed zoonotic severe acute respiratory syndrome coronavirus. J. Virol..

[11] Gaunt E.R., Hardie A., Claas E.C.J., Simmonds P., Templeton K.E. (2010). Epidemiology and clinical presentations of the four Human Coronaviruses 229E, HKU1, NL63, and OC43 detected over 3 years using a novel multiplex real-time PCR method. J. Clin. Microbiol..

[12] Woldemeskel B.A., Kwaa A.K., Garliss C.C., Laeyendecker O., Ray S.C., Blankson J.N. (2020). Healthy donor T cell responses to common cold coronaviruses and SARS-CoV-2. J. Clin. Investig..

[13] Abdul-Rasool S., Fielding B.C. (2010). Understanding human Coronavirus. Open Virol. J..

[14] Esper F., Weibel C., Ferguson D., Landry M.L., Kahn J.S. (2006). Coronavirus HKU1 infection in the United States. Emerg. Infect. Dis..

[15] Pene F., Merlat A., Vabret A., Rozenberg F., Buzyn A., Dreyfus F., Cariou A., Freymuth F., Lebon P. (2003). Coronavirus 229E-related pneumonia in immunocompromised patients. Clin. Infect. Dis..

[16] Jordan P.C., Stevens S.K., Deval J. (2018). Nucleosides for the treatment of respiratory RNA virus infections. Antivir. Chem. Chemother..

[17] Cevik M., Tate M., Lloyd O., Maraolo A.E., Schafers J., Ho A. (2020). SARS-CoV-2, SARS-CoV-1 and MERS-CoV viral load dynamics, duration of viral shedding and infectiousness: A living systematic review and meta-analysis. SSRN Electron. J..

[18] Andersen K.G., Rambaut A., Lipkin W.I., Holmes E.C., Garry R.F. (2020). The proximal origin of SARS-CoV-2. Nat. Med..

[19] Rabaan A.A., Al-Ahmed S.H., Haque S., Sah R., Tiwari R., Malik Y.S., Dhama K., Yatoo M.I., Bonilla-Aldana D.K., Rodriguez-Morales A.J. (2020). SARS-CoV-2, SARS-CoV, and MERS-COV: A comparative overview. Infez. Med..

[20] Peeri N.C., Shrestha N., Rahman M.S., Zaki R., Tan Z., Bibi S., Baghbanzadeh M., Aghamohammadi N., Zhang W., Haque U. (2020). The SARS, MERS and novel coronavirus (COVID-19) epidemics, the newest and biggest global health threats: What lessons have we learned?. Int. J. Epidemiol..

[21] Gordon D.E., Hiatt J., Bouhaddou M., Rezelj V.V., Ulferts S., Braberg H., Jureka A.S., Obernier K., Guo J.Z., Batra J. (2020). Comparative host-coronavirus protein interaction networks reveal pan-viral disease mechanisms. Science.

[22] Heurich A., Hofmann-Winkler H., Gierer S., Liepold T., Jahn O., Pohlmann S. (2013). TMPRSS2 and ADAM17 Cleave ACE2 differentially and only proteolysis by TMPRSS2 augments entry driven by the severe acute respiratory syndrome coronavirus spike protein. J. Virol..

[23] Belouzard S., Millet J.K., Licitra B.N., Whittaker G.R. (2012). Mechanisms of coronavirus cell entry mediated by the viral spike protein. Viruses.

[24] Hulswit R.J.G., de Haan C.A.M., Bosch B.J. (2016). Coronavirus spike protein and tropism changes. Adv. Virus Res..

[25] Kirchdoerfer R.N., Cottrell C.A., Wang N., Pallesen J., Yassine H.M., Turner H.L., Corbett K.S., Graham B.S., McLellan J.S., Ward A.B. (2016). Pre-fusion structure of a human coronavirus spike protein. Nature.

[26] Pillay T.S. (2020). Gene of the month: The 2019-nCoV/SARS-CoV-2 novel coronavirus spike protein. J. Clin. Pathol..

[27] Xia S., Liu M., Wang C., Xu W., Lan Q., Feng S., Qi F., Bao L., Du L., Liu S. (2020). Inhibition of SARS-CoV-2 (previously 2019-nCoV) infection by a highly potent pan-coronavirus fusion inhibitor targeting its spike protein that harbors a high capacity to mediate membrane fusion. Cell Res..

[28] Roviello V., Musumeci D., Mokhir A., Roviello G.N. (2021). Evidence of protein binding by a nucleopeptide based on a thymine-decorated L-diaminopropanoic acid through CD and in silico studies. Curr. Med. Chem..

[29] Kim C.-H. (2020). SARS-CoV-2 evolutionary adaptation toward host entry and recognition of receptor O-acetyl sialylation in virus–host interaction. Int. J. Mol. Sci..

[30] Artese A., Svicher V., Costa G., Salpini R., Di Maio V.C., Alkhatib M., Ambrosio F.A., Santoro M.M., Assaraf Y.G., Alcaro S. (2020). Current status of antivirals and druggable targets of SARS CoV-2 and other human pathogenic coronaviruses. Drug Resist. Updates.

[31] Schoeman D., Fielding B.C. (2019). Coronavirus envelope protein: Current knowledge. Virol. J..

[32] Chen B., Tian E.-K., He B., Tian L., Han R., Wang S., Xiang Q., Zhang S., El Arnaout T., Cheng W. (2020). Overview of lethal human coronaviruses. Signal Transduct. Target. Ther..

[33] Cucinotta D., Vanelli M. (2020). WHO declares COVID-19 a pandemic. Acta Biomed..

[34] Mercatelli D., Holding A.N., Giorgi F.M. (2020). Web tools to fight pandemics: The COVID-19 experience. Brief. Bioinform..

[35] Arthi V., Parman J. (2020). Disease, downturns, and wellbeing: Economic history and the long-run impacts of COVID-19. Explorat. Econ. History.

[36] Roviello V., Roviello G.N. (2020). Lower COVID-19 mortality in Italian forested areas suggests immunoprotection by Mediterranean plants. Environ. Chem. Lett..

[37] Ibn-Mohammed T., Mustapha K.B., Godsell J., Adamu Z., Babatunde K.A., Akintade D.D., Acquaye A., Fujii H., Ndiaye M.M., Yamoah F.A. (2021). A critical analysis of the impacts of COVID-19 on the global economy and ecosystems and opportunities for circular economy strategies. Resour. Conserv. Recycl..

[38] Naveja J.J., Madariaga-Mazón A., Flores-Murrieta F., Granados-Montiel J., Maradiaga-Ceceña M., Alaniz V.D., Maldonado-Rodriguez M., García-Morales J., Senosiain-Peláez J.P., Martínez-Mayorga K. (2020). Union is strength: Antiviral and anti-inflammatory drugs for COVID-19. Drug Disc. Today.

[39] Zhang W., Zhao Y., Zhang F., Wang Q., Li T., Liu Z., Wang J., Qin Y., Zhang X., Yan X. (2020). The use of anti-inflammatory drugs in the treatment of people with severe coronavirus disease 2019 (COVID-19): The Perspectives of clinical immunologists from China. Clin. Immunol..

[40] Costanzo M., De Giglio M.A.R., Roviello G.N. (2020). SARS CoV-2: Recent reports on antiviral therapies Based on lopinavir/ritonavir, darunavir/umifenovir, hydroxychloroquine, remdesivir, favipiravir and other drugs for the treatment of the new coronavirus. Curr. Med. Chem..

[41] Singh T.U., Parida S., Lingaraju M.C., Kesavan M., Kumar D., Singh R.K. (2020). Drug repurposing approach to fight COVID-19. Pharmacol. Rep..

[42] Borbone N., Piccialli G., Roviello G.N., Oliviero G. (2021). Nucleoside Analogs and Nucleoside Precursors as Drugs in the Fight against SARS-CoV-2 and Other Coronaviruses. Molecules.

[43] Ang L., Lee H.W., Choi J.Y., Zhang J., Lee M.S. (2020). Herbal medicine and pattern identification for treating COVID-19: A rapid review of guidelines. Integr. Med. Res..

[44] Li Y., Liu X., Guo L., Li J., Zhong D., Zhang Y., Clarke M., Jin R. (2020). Traditional Chinese herbal medicine for treating novel coronavirus (COVID-19) pneumonia: Protocol for a systematic review and meta-analysis. Syst. Rev..

[45] Ang L., Lee H.W., Kim A., Lee J.A., Zhang J., Lee M.S. (2020). Herbal medicine for treatment of children diagnosed with COVID-19: A review of guidelines. Complement. Ther. Clin. Pract..

[46] Shahrajabian M.H., Sun W., Shen H., Cheng Q. (2020). Chinese herbal medicine for SARS and SARS-CoV-2 treatment and prevention, encouraging using herbal medicine for COVID-19 outbreak. Acta Agric. Scand. Sect. B Soil Plant Sci..

[47] Lee D.Y., Li Q.Y., Liu J., Efferth T. (2021). Traditional Chinese herbal medicine at the forefront battle against COVID-19: Clinical experience and scientific basis. Phytomedicine.

[48] Wei H., Liu S., Liao Y., Ma C., Wang D., Tong J., Feng J., Yi T., Zhu L. (2019). A systematic review of the medicinal potential of mulberry in treating diabetes mellitus. Am. J. Chin. Med..

[49] Ng K.-W., Cao Z.-J., Chen H.-B., Zhao Z.-Z., Zhu L., Yi T. (2017). Oolong tea: A critical review of processing methods, chemical composition, health effects, and risk. Crit. Rev. Food Sci. Nutr..

[50] Yi T., Chen H.-B., Zhao Z.-Z., Yu Z.-L., Jiang Z.-H. (2011). Comparison of the chemical profiles and anti-platelet aggregation effects of two “Dragon’s Blood” drugs used in traditional Chinese medicine. J. Ethnopharmacol..

[51] Yi T., Tang Y., Zhang J., Zhao Z., Yang Z., Chen H. (2012). Characterization and determination of six flavonoids in the ethnomedicine “Dragon’s Blood” by UPLC-PAD-MS. Chem. Cent. J..

[52] Xue Y., Zhu L., Yi T. (2017). Fingerprint analysis of Resina Draconis by ultra-performance liquid chromatography. Chem. Cent. J..

[53] Zheng G.-Q., Kenney P.M., Lam L.K. (1992). Sesquiterpenes from clove (*Eugenia caryophyllata*) as potential anticarcinogenic agents. J. Nat. Prod..

[54] Bhowmik D., Kumar K.S., Yadav A., Srivastava S., Paswan S., Dutta A.S. (2012). Recent trends in Indian traditional herbs *Syzygium aromaticum* and its health benefits. J. Pharm. Phytochem..

[55] Cortés-Rojas D.F., de Souza C.R.F., Oliveira W.P. (2014). Clove (*Syzygium aromaticum*): A precious spice. Asian Pac. J. Trop. Biomed..

[56] Aisha A.F., Abu-Salah K.M., Alrokayan S.A., Siddiqui M.J., Ismail Z., Majid A.M.S.A. (2012). *Syzygium aromaticum* extracts as good source of betulinic acid and potential anti-breast cancer. Rev. Bras. Farm..

[57] Prashar A., Locke I.C., Evans C.S. (2006). Cytotoxicity of clove (*Syzygium aromaticum*) oil and its major components to human skin cells. Cell Prolif..

[58] Banerjee S., Panda C.K., Das S. (2006). Clove (*Syzygium aromaticum* L.), a potential chemopreventive agent for lung cancer. Carcinogenesis.

[59] Aisha A., Nassar Z., Siddiqui M., Abu-Salah K., Alrokayan S., Ismail Z., Abdul Majid A. (2011). Evaluation of antiangiogenic, cytotoxic and antioxidant effects of *Syzygium aromaticum* L. extracts. Asian J. Biol. Sci..

[60] Ogata M., Hoshi M., Urano S., Endo T. (2000). Antioxidant activity of eugenol and related monomeric and dimeric compounds. Chem. Pharm. Bull..

[61] Darshan S., Doreswamy R. (2004). Patented antiinflammatory plant drug development from traditional medicine. Phytother. Res..

[62] Miyazawa M., Hisama M. (2001). Suppression of chemical mutagen-induced SOS response by alkylphenols from clove (*Syzygium aromaticum*) in the Salmonella typhimurium TA1535/pSK1002 umu test. J. Agric. Food Chem..

[63] Vijayasteltar L., Nair G.G., Maliakel B., Kuttan R., Krishnakumar I. (2016). Safety assessment of a standardized polyphenolic extract of clove buds: Subchronic toxicity and mutagenicity studies. Toxicol. Rep..

[64] Ogunwande I., Olawore N., Ekundayo O., Walker T., Schmidt J., Setzer W. (2005). Studies on the essential oils composition, antibacterial and cytotoxicity of *Eugenia uniflora* L.. Int. J. Aromather..

[65] Udensi U.K., Tchounwou P.B. (2017). Potassium homeostasis, oxidative stress, and human disease. Int. J. Clin. Exp. Physiol..

[66] Horsburgh M.J., Wharton S.J., Karavolos M., Foster S.J. (2002). Manganese: Elemental defence for a life with oxygen. Trends Microbiol..

[67] Fang S., Thomas R.M., Conklin J.L., Oberley L.W., Christensen J. (1995). Co-localization of manganese superoxide dismutase and NADH diaphorase. J. Histochem. Cytochem..

[68] Cao Y., Qin Y., Bruist M., Gao S., Wang B., Wang H., Guo X. (2015). Formation and dissociation of the interstrand i-motif by the sequences d(XnC4Ym) monitored with electrospray ionization mass spectrometry. J. Am. Soc. Mass Spectrom..

[69] Wilson J.X. (2009). Mechanism of action of vitamin C in sepsis: Ascorbate modulates redox signaling in endothelium. Biofactors.

[70] Bagchi D., Garg A., Krohn R., Bagchi M., Tran M., Stohs S. (1997). Oxygen free radical scavenging abilities of vitamins C and E, and a grape seed proanthocyanidin extract in vitro. Res. Commun. Mol. Pathol. Pharmacol..

[71] Pino J.A., Marbot R., Agüero J., Fuentes V. (2001). Essential oil from buds and leaves of clove (*Syzygium aromaticum* (L.) Merr. et Perry) grown in Cuba. J. Essent. Oil Res..

[72] Raina V., Srivastava S., Aggarwal K., Syamasundar K., Kumar S. (2001). Essential oil composition of *Syzygium aromaticum* leaf from Little Andaman, India. Flavour Fragr. J..

[73] Zachariah T., Krishnamoorthy B., Rema J., Mathew P. (2005). Oil constituents in bud and pedicel of clove (*Syzygium aromaticum*). Ind. Perf..

[74] Mittal M., Gupta N., Parashar P., Mehra V., Khatri M. (2014). Phytochemical evaluation and pharmacological activity of *Syzygium aromaticum*: A comprehensive review. Int. J. Pharm. Pharm. Sci..

[75] Jimoh S.O., Arowolo L.A., Alabi K.A. (2017). Phytochemical screening and antimicrobial evaluation of *Syzygium aromaticum* extract and essential oil. Int. J. Curr. Microbiol. Appl. Sci..

[76] El Ghallab Y., Al Jahid A., Eddine J.J., Said A.A.H., Zarayby L., Derfoufi S. (2020). *Syzygium aromaticum* L.: Phytochemical investigation and comparison of the scavenging activity of essential oil, extracts and eugenol. Adv. Tradit. Med..

[77] Begum S., Siddiqui B.S., Khatoon R., Aftab F. (2014). Phytochemical studies on *Syzygium aromaticum* Linn. J. Chem. Soc. Pak..

[78] Chaieb K., Hajlaoui H., Zmantar T., Kahla-Nakbi A.B., Rouabhia M., Mahdouani K., Bakhrouf A. (2007). The chemical composition and biological activity of clove essential oil, *Eugenia caryophyllata* (*Syzigium aromaticum* L. Myrtaceae): A short review. Phytother. Res..

[79] Gopalakrishnan N., Narayanan C., Mathew A. (1984). Sesquiterpene hydrocarbons from clove oil. Lebensmittel-Wissenschaft+ Technol..

[80] Zhao C., Qiao X., Shao Q., Hassan M., Ma Z. (2020). Evolution of the lignin chemical structure during the bioethanol production process and its inhibition to enzymatic hydrolysis. Energy Fuels.

[81] Frohlich P.C., Santos K.A., Palú F., Cardozo-Filho L., da Silva C., da Silva E.A. (2019). Evaluation of the effects of temperature and pressure on the extraction of eugenol from clove (*Syzygium aromaticum*) leaves using supercritical CO_2_. J. Supercrit. Fluids.

[82] Bahramsoltani R., Rahimi R. (2020). An evaluation of traditional Persian medicine for the management of SARS-CoV-2. Front. Pharmacol..

[83] Öztürk A., Özbek H. (2005). The anti-inflammatory activity of Eugenia caryophyllata essential oil: An animal model of anti-inflammatory activity. Eur. J. Gen Med..

[84] Tanko Y., Mohammed A., Okasha M., Umah A., Magaji R. (2008). Anti-nociceptive and anti-inflammatory activities of ethanol extract of *Syzygium aromaticum* flower bud in wistar rats and mice. Afr. J. Trad. Complement. Altern. Med..

[85] Martin S., Padilla E., Ocete M., Galvez J., Jimenez J., Zarzuelo A. (1993). Anti-inflammatory activity of the essential oil of *Bupleurum fruticescens*. Planta Med..

[86] Rho H.S., Ghimeray A.K., Yoo D.S., Ahn S.M., Kwon S.S., Lee K.H., Cho D.H., Cho J.Y. (2011). Kaempferol and kaempferol rhamnosides with depigmenting and anti-inflammatory properties. Molecules.

[87] García-Mediavilla V., Crespo I., Collado P.S., Esteller A., Sánchez-Campos S., Tuñón M.J., González-Gallego J. (2007). The anti-inflammatory flavones quercetin and kaempferol cause inhibition of inducible nitric oxide synthase, cyclooxygenase-2 and reactive C-protein, and down-regulation of the nuclear factor kappaB pathway in Chang Liver cells. Eur. J. Pharmacol..

[88] Jnawali H.N., Lee E., Jeong K.-W., Shin A., Heo Y.-S., Kim Y. (2014). Anti-inflammatory activity of rhamnetin and a model of its binding to c-Jun NH2-terminal kinase 1 and p38 MAPK. J. Nat. Prod..

[89] Novo Belchor M., Hessel Gaeta H., Fabri Bittencourt Rodrigues C., Ramos da Cruz Costa C., de Oliveira Toyama D., Domingues Passero L.F., Dalastra Laurenti M., Hikari Toyama M. (2017). Evaluation of rhamnetin as an inhibitor of the pharmacological effect of secretory phospholipase A2. Molecules.

[90] Daniel A.N., Sartoretto S.M., Schmidt G., Caparroz-Assef S.M., Bersani-Amado C.A., Cuman R.K.N. (2009). Anti-inflammatory and antinociceptive activities A of eugenol essential oil in experimental animal models. Rev. Bras. Farm..

[91] Magalhães C.B., Riva D.R., DePaula L.J., Brando-Lima A., Koatz V.L.G., Leal-Cardoso J.H., Zin W.A., Faffe D.S. (2010). In vivo anti-inflammatory action of eugenol on lipopolysaccharide-induced lung injury. J. Appl. Physiol..

[92] Chniguir A., Zioud F., Marzaioli V., El-Benna J., Bachoual R. (2019). *Syzygium aromaticum* aqueous extract inhibits human neutrophils myeloperoxidase and protects mice from LPS-induced lung inflammation. Pharm. Biol..

[93] Nassan M., Mohamed E., Abdelhafez S., Ismail T. (2015). Effect of clove and cinnamon extracts on experimental model of acute hematogenous pyelonephritis in albino rats: Immunopathological and antimicrobial study. Int. J. Immunopathol. Pharmacol..

[94] Su H., Yang M., Wan C., Yi L.-X., Tang F., Zhu H.-Y., Yi F., Yang H.-C., Fogo A.B., Nie X. (2020). Renal histopathological analysis of 26 postmortem findings of patients with COVID-19 in China. Kidney Int..

[95] Carrasco F.R., Schmidt G., Romero A.L., Sartoretto J.L., Caparroz-Assef S.M., Bersani-Amado C.A., Cuman R.K.N. (2009). Immunomodulatory activity of *Zingiber officinale Roscoe*, *Salvia officinalis* L. and *Syzygium aromaticum* L. essential oils: Evidence for humor-and cell-mediated responses. J. Pharm. Pharmacol..

[96] Saeed S.A., Gilani A.H. (1994). Antithrombotic activity of clove oil. J. Pak. Med. Assoc..

[97] Yang Y.-Y., Lee M.-J., Lee H.-S., Park W.-H. (2011). Screening of antioxidative, anti-platelet aggregation and anti-thrombotic effects of clove extracts. J. Physiol. Pathol. Korean Med..

[98] Rasheed A., Laekeman G., Totte J., Vlietinck A., Herman A. (1984). Eugenol and prostaglandin biosynthesis. N. Engl. J. Med..

[99] Srivastava K. (1993). Antiplatelet principles from a food spice clove (*Syzgium aromaticum* L). Prostag. Leukot. Essent. Fat. Acids.

[100] Im LEE J., Lee H.S., Jun W.J., Yu K.W., Shin D.H., Hong B.S., Cho H.Y., Yang H.C. (2001). Purification and Characterization of Antithrombotics from Syzygium aromaticum (L.) MERR. & PERRY. Biol. and Pharm. Bull..

[101] Tragoolpua Y., Jatisatienr A. (2007). Anti-herpes simplex virus activities of *Eugenia caryophyllus* (Spreng.) Bullock & SG Harrison and essential oil, eugenol. Phytother. Res..

[102] Hussein G., Miyashiro H., Nakamura N., Hattori M., Kakiuchi N., Shimotohno K. (2000). Inhibitory effects of Sudanese medicinal plant extracts on hepatitis C virus (HCV) protease. Phytother. Res..

[103] Benencia F., Courreges M. (2000). In vitro and in vivo activity of eugenol on human herpesvirus. Phytother. Res..

[104] Dai J.-P., Zhao X.-F., Zeng J., Wan Q.-Y., Yang J.-C., Li W.-Z., Chen X.-X., Wang G.-F., Li K.-S. (2013). Drug screening for autophagy inhibitors based on the dissociation of Beclin1-Bcl2 complex using BiFC technique and mechanism of eugenol on anti-influenza A virus activity. PLoS ONE.

[105] Lane T., Anantpadma M., Freundlich J.S., Davey R.A., Madrid P.B., Ekins S. (2019). The natural product eugenol is an inhibitor of the ebola virus in vitro. Pharm. Res..

[106] Kurokawa M., Hozumi T., Basnet P., Nakano M., Kadota S., Namba T., Kawana T., Shiraki K. (1998). Purification and Characterization of Eugeniin as an Anti-herpesvirus Compound from *Geum japonicum* and *Syzygium aromaticum*. J. Pharmacol. Exp. Ther..

[107] Saleem H.N., Batool F., Mansoor H.J., Shahzad-ul-Hussan S., Saeed M. (2019). Inhibition of dengue virus protease by Eugeniin, Isobiflorin, and Biflorin Isolated from the Flower Buds of *Syzygium aromaticum* (Cloves). ACS Omega.

[108] Kanyinda J.N.M. (2020). Coronavirus (COVID-19): A protocol for prevention and treatment (Covalyse^®^). Eur. J. Med. Health Sci..

[109] Samaddar A., Gadepalli R., Nag V.L., Misra S. (2020). The enigma of low COVID-19 fatality rate in India. Front. Genet..

[110] Rhodes J.M., Subramanian S., Laird E., Kenny R.A. (2020). Low population mortality from COVID-19 in countries south of latitude 35 degrees North supports vitamin D as a factor determining severity. Aliment. Pharmacol. Ther..

[111] Singh N.A., Kumar P., Kumar N. (2021). Spices and herbs: Potential antiviral preventives and immunity boosters during COVID-19. Phytother. Res..

[112] Chaachouay N., Douira A., Zidane L. (2021). COVID-19, prevention and treatment with herbal medicine in the herbal markets of Salé Prefecture, North-Western Morocco. Eur. J. Integrat. Med..

[113] Pandey P., Singhal D., Khan F., Arif M. (2020). An in silico screening on *Piper nigrum*, *Syzygium aromaticum* and *Zingiber officinale* roscoe derived compounds against SARS-CoV-2: A drug repurposing approach. Biointerface Res. Appl. Chem..

[114] Joshi T., Joshi T., Sharma P., Mathpal S., Pundir H., Bhatt V., Chandra S. (2020). In silico screening of natural compounds against COVID-19 by targeting Mpro and ACE2 using molecular docking. Eur. Rev. Med. Pharmacol. Sci..

[115] Rehman M., AlAjmi M.F., Hussain A. (2020). Natural compounds as inhibitors of SARS-CoV-2 main protease (3CLpro): A molecular docking and simulation approach to combat COVID-19. Curr. Pharm. Des..

